# Stimulants and Narcotics

**Published:** 1872-05

**Authors:** 


*Stimulants and Narcotics.* Medically, Philosophically and Morally considered. By George M. Beard, M. D. New York, G. P. Putnam & Sons, 1871.

These two little books form numbers three and four of Putnams handy book series, and are composed of matters of interest to the popular mind, which are not usually treated of in the literature of the day.

Eating and Drinking, gives a history of the different kinds of food, and their character. the manner of preparing them, together with rules for diet in health and disease. Stimulants and Narcotics, is the title of a little work which treats those subjects in all their varied points of view. Intemperance and the various means proposed for its cure are considered to some length; and are of much interest to the practitioner as well as to the general reader. The concluding sentence of the book. "The future of Temperance must inevitably share in the future of civilization" expresses the true idea in regard to the subject. Educate the people to higher and nobler aspirations and there will be less intemperance.

